# Curcumin protects brain from oxidative stress through inducing expression of UCP2 in chronic cerebral hypoperfusion aging-rats

**DOI:** 10.1186/1750-1326-7-S1-S10

**Published:** 2012-02-07

**Authors:** Li Liu, Peng Zhang, Yu Li, Gang Yu

**Affiliations:** 1 Department of Pathology, Chongqing Medical University, Chongqing, 400016, China; 2 Institute of Neuroscience, Chongqing Medical University, Chongqing, 400016, China; 3 Chongqing Key Laboratory of Neurobiology, Chongqing Medical University, Chongqing, 400016, China; 4 Department of Neurology, The First Affiliated Hospital , Chongqing Medical University, Chongqing, 400016, China

## Background

Chronic cerebral ischemia is caused by the long-term cerebral hypoperfusion, which is a common pathological process that usually occurs in conditions such as Alzheimer’s disease and vascular dementia, both of which are characterized by cognitive impairment. Oxidative stress plays an important role in nerve cell damage and cognitive dysfunction induced by chronic cerebral ischemia, but the UCP2 (The uncoupling protein 2) plays an important role in inhibiting oxidative stress. This study aims to observe the antioxidation of Curcumin on chronic cerebral ischemia model in rats and investigate change of UCP2 induced by Curcumin.

## Methods

The chronic cerebral ischemia was produced in male Sprague-Dawley rats by permanent occlusion of bilateral common carotid arteries (2VO). Animals were randomly divided into 5 groups: normal control group, sham-operated group, 2VO+DMSO group, 2VO+Curcumin 100mg/kg group, 2VO+Curcumin 50mg/kg group. After surgery, all animals were injected intraperitoneally with DMSO solution of Curcumin or a same volume of normal DMSO. Each group was injected once daily for four consecutive weeks. The spatial learning and memory ability was tested by Morris water maze after administration for 28d. After the completion of the behavioral testing, rats were sacrificed. Hippocampus was used to spectrophotometrically determine the level of MDA and the activity of SOD. The pathological changes in the hippocampus CA1 area were observed with hematoxylin and eosin (HE) staining and Nissl staining. The expressions of UCP2 protein in hippocampus were detected by immunohistochemistry.

## Results

The results displayed that Curcumin significantly improved the spatial learning and memory and attenuated pathological change in the hippocampus CA1 area. The level of MDA decreased, but the activity of SOD increased, and the changes were in a dose-dependent manner (p<0.05). In the hippocampus the expression of UCP2 protein significantly increased after treated with Curcumin.

## Conclusion

Curcumin has an obvious neuroprotective effect on brain injury induced by chronic cerebral ischemia and can inhibit oxidative stress induced by ischemia. It is the main performance that improving the spatial learning and memory, attenuating pathological change, decreasing the level of MDA, increasing the activity of SOD and inducing HO-1 protein expression. Our data demonstrated that the neuroprotective effect of Curcumin involved in increasing the protein levels of UCP2 and inhibiting oxidative stress induced by ischemia.

